# NKp46^+^ Innate Lymphoid Cells Dampen Vaginal CD8 T Cell Responses following Local Immunization with a Cholera Toxin-Based Vaccine

**DOI:** 10.1371/journal.pone.0143224

**Published:** 2015-12-02

**Authors:** Carmelo Luci, Selma Bekri, Franck Bihl, Jonathan Pini, Pierre Bourdely, Kelly Nouhen, Angélique Malgogne, Thierry Walzer, Véronique M. Braud, Fabienne Anjuère

**Affiliations:** 1 Université de Nice Sophia Antipolis, Institut de Pharmacologie Moléculaire et Cellulaire, Sophia Antipolis, France; 2 Centre National de la Recherche Scientifique, Unité Mixte de Recherche 7275, Sophia Antipolis, France; 3 Institut National de la Santé et de la Recherche Médicale, Institut de Pharmacologie Moléculaire et Cellulaire, France; 4 Université de Nice Sophia Antipolis, Centre National de la Recherche Scientifique, Unité Mixte de Recherche 7370, Laboratoire de PhysioMédecine Moléculaire, Nice, France; 5 Université de Lyon 1, Ecole Normale Supérieure de Lyon, Institut National de la Santé et de la Recherche Médicale U1111, Centre National de la Recherche Scientifique, Unité Mixte de Recherche 5308, Centre International de recherche en infectiologie, Lyon, France; Harvard Medical School, UNITED STATES

## Abstract

Innate and adaptive immune cells work in concert to generate efficient protection at mucosal surface. Vaginal mucosa is an epithelial tissue that contains innate and adaptive immune effector cells. Our previous studies demonstrated that vaginal administration of Cholera toxin -based vaccines generate antigen-specific CD8 T cells through the stimulation of local dendritic cells (DC). Innate lymphoid cells (ILC) are a group of lymphocytes localized in epithelial tissues that have important immune functions against pathogens and in tissue homeostasis. Their contribution to vaccine-induced mucosal T cell responses is an important issue for the design of protective vaccines. We report here that the vaginal mucosa contains a heterogeneous population of NKp46^+^ ILC that includes conventional NK cells and ILC1-like cells. We show that vaginal NKp46^+^ ILC dampen vaccine-induced CD8 T cell responses generated after local immunization. Indeed, *in vivo* depletion of NKp46^+^ ILC with anti-NK1.1 antibody or NKG2D blockade increases the magnitude of vaginal OVA-specific CD8 T cells. Furthermore, such treatments also increase the number of DC in the vagina. NKG2D ligands being expressed by vaginal DC but not by CD8 T cells, these results support that NKp46^+^ ILC limit mucosal CD8 T cell responses indirectly through the NKG2D-dependent elimination of vaginal DC. Our data reveal an unappreciated role of NKp46^+^ ILC in the regulation of mucosal CD8 T cell responses.

## Introduction

The establishment of protective immunity at the entry site of sexually transmitted pathogens involves long-lasting tissue-associated memory T and B lymphocytes and is required to efficiently block the systemic spreading of those pathogens [1]. Genital protective B and T cell immunity is better achieved by mucosal rather than by parenteral immunization and antigens need to be admixed with appropriate adjuvants to develop efficient mucosal vaccines. A better understanding of the cellular mechanisms induced after intravaginal vaccination may help optimizing vaccines against sexually transmitted infection [2].

The *Vibrio cholerae* toxin (CT) is a potent mucosal adjuvant widely used to understand the cellular and molecular events induced by mucosal immunization [3, 4]. CT has an AB5 structure composed of two distinct subunits: a single toxic A subunit (CTA) and a nontoxic homopentameric B subunit (CTB). CTB has a strong affinity for GM1 gangliosides that are present on all mammalian nucleated cells and are responsible for the anchor of the toxin onto host cells. CTB, which can be produced independently of the CTA moiety, constitutes an efficient antigen-delivery system for covalently linked proteins and polysaccharides [5]. We have shown that the intravaginal or the sublingual application of antigens conjugated to CTB and co-administered with CT induces antigen-specific IgA antibody secreting cells (ASCs) and cytotoxic CD8 T cells in the female genital tract [6–9]. Moreover, the covalent linking of antigens to CTB is mandatory to favor their processing by mucosa derived dendritic cells (DC) and their delivery into the MHC class I pathway, thus leading to the generation of cytotoxic CD8 T cell responses [6, 10]. Nevertheless, the contribution of other innate leukocytes present at mucosal sites in the regulation of those responses remains to be explored.

Innate lymphoid cells (ILC) are a family of specialized lymphocytes of the innate immune system preferentially localized in epithelial tissues (intestine, skin, lung, uterus). They serve as an early source of cytokines to regulate mucosal immune responses but also to ensure tissue repair and tissue homeostasis [11]. ILC family comprises the well-described conventional cytotoxic natural killer cells (NK cells) that patrol lymphoid and non-lymphoid organs to discriminate and eliminate stressed cells (*i*.*e*. infected and tumor cells) as well as other ILC subsets. The ILC population shares expression of cell surface markers such as the IL-7 receptor-α (CD127) or IL-2 receptor-α (CD25) but they are distinct in terms of cytokine production: group 1 ILC (ILC1) produce interferon-γ (IFN-γ) and tumor necrosis factor-α (TNF-α); group 2 ILC (ILC2) produce IL-5 and IL-13 and group 3 ILC (ILC3, that include lymphoid tissue inducer cells) produce IL-17 and IL-22 [12–14]. NKp46 was originally used to identify NK cells as it is expressed from early to late stages of NK cell differentiation [15]. This holds true in lymphoid organs but not in non lymphoid tissues where NKp46 also identifies some ILC1 and ILC3 subsets [13, 16, 17]. Moreover, ILC subsets are also defined on the basis of their differential requirements for transcription factors (TF) involved in their development and/or survival. Whereas NK cells required both T-bet and Eomes (Eomesodermine), ILC1 need only T-bet for their development. On the other hand, the TF GATA3 and RORα are necessary for ILC2 maintenance and RORγt is indispensable for all ILC3 subsets development [18, 19].

It remains unknown whether the vaginal mucosa contains these recently described ILC subsets and only very few studies focused on the contribution of conventional NK cells in vaginal immunity. In NK cell deficient IL15^-/-^ and RAG-2^-/-^ / γc ^-/-^ mice, intravaginal infection with Herpes Simplex Virus 2 (HSV 2) resulted in impaired innate immune response [20]. Moreover, depletion of NK1.1^+^ NK cells in the vaginal tissue of wild type C57BL/6 mice using the depleting anti-NK1.1 antibody resulted in increased sensitivity of these mice to vaginal HSV 2 infection [21]. NK1.1 is also expressed by the ILC1 subset in mice [18, 22]. Therefore, a detailed characterization of vaginal ILC and their contribution to vaginal immunity induced by local vaccination is needed to better understand the regulation of adaptive immune responses in the female genital tract.

Here, we show that NKp46^+^ ILC in the mouse vaginal mucosa encompass Eomes^+^ NKp46^+^ NK1.1^+^ CD49a^-^ NK cells and Eomes^-^ NKp46^+^ NK1.1^+^ CD49a^+^ILC1-like cells. We used a mouse model of vaccination based on the vaginal administration of a subunit vaccine composed of ovalbumin covalently linked to CTB and co-administered with CT (CTBOVA+CT) previously described to generate vaginal effector CD8 T cells [6]. In this experimental model, we demonstrated that vaginal NKp46^+^ ILC negatively regulate vaccine-induced CD8 T lymphocytes. We propose that NKp46^+^ ILC control vaginal CD8 T cell expansion presumably by limiting the number of antigen-presenting dendritic cells via a NKG2D dependent mechanism.

## Material and Methods

### Animals

Six to ten weeks-old female C57BL/6 mice were purchased from Charles River Laboratories (France).

### Ethics statement

Animals were housed in isolators under pathogen-free conditions according to institutional guidelines. Anesthesia was performed using Ketamine and Xylazine to minimize suffering. Experiments were approved by an ethical committee for animal experimentation: Centre National de Reflexion Ethique sur l'Experimentation Animal: reference # CIEPAL n°NCE/2015-220.

### Synthesis of cholera toxin-based vaccine

Cholera toxin (CT) was obtained from List Biologicals Laboratories (USA) and recombinant CTB from Crucell AB (Sweden). Albumin Chicken Egg (ovalbumin, OVA Grade VII, Sigma-Aldrich, France) was conjugated to CTB as described earlier [6].

### Immunizations and injections of antibodies

C57BL/6 mice synchronized by Depoprovera® treatment were immunized under anesthesia by vaginal application of CTBOVA (4μg) + CT (1μg) in PBS at days 0, 14, and 21. NKp46^+^ ILC cells were depleted after intraperitoneal (i.p) injection of 200μL of anti-NK1.1 mAb (PK136 clone, 1/20 dilution of ascite fluid) at days -9 and 2 before each immunization. Such protocol insured at least a 98% depletion of NKp46^+^CD3^-^ ILC cells in spleen, genital lymph nodes and vagina without affecting invariant natural killer T (iNKT) cells. NKG2D/NKG2D ligands blockade was performed by i.p injection of 500μg of mAb anti-NKG2D (CX5) at days -1 and +1 before and after each immunization. For dendritic cells tracking experiments, mice were immunized once with CTBA488 (40μg) and CT (1μg) and were sacrificed after 7 days.

### Cell preparation

Vaginas from PBS-perfused mice were excised and cells dispersed by collagenase A (1mg/mL) and DNase I (0,2mg/mL) (Roche Diagnostics) treatment at 37°C for 20 minutes. Spleen and GLN cells were processed similarly to ensure that enzymatic treatment does not alter the surface expression of markers analyzed and the functions of cells. Small intestine NKp46 ILC were prepared as previously described [16]. Dendritic cells were purified from GLN and vaginas as previously described [6]. NKp46 ILC numbers per organ were determined by multiplying counted total live cells by the percentage of gated CD45^+^ NKp46^+^ CD3^-^ cells obtained by flow cytometry.

### Flow cytometry

Cells were preincubated with anti FcγRII/III mAb (2.4G2) for 10 min at 4°C to block Fc receptors. Unless indicated, antibodies were purchased from BD Biosciences. Cell suspensions were analyzed by flow cytometry using CD122 (TM-Beta 1), NK1.1 (PK136), NKG2D (CX5, eBiosciences), NKG2A/C/E (20d5), CD107a (1D4B), CD27 (LG.3A10), IFN-γ (XMG1.2), NK1.1 (PK136), CD3 (500A2), CD11b (M1/70), NKp46 (29A1.4), CD45 (30-F11), CD49b (DX5), CD49a (Ha31/8), Ia-Ie (2G9), CD11c (HL3), Eomes (Dan11mag, eBiosciences), Tbet (eBio4B10, eBiosciences), RORγt (AFKJ-9; eBiosciences), pan-specific Rae1 (186107, R&D systems), MULT1 (237104, R and D systems), TRAIL (N2B2, eBiosciences), TNFα (MPX-XT22) antibodies conjugated to different fluorochromes. Frequency of OVA-specific CD8 T cells was analyzed after staining with anti-CD8 (53–6.7), anti-CD3 (500A2) and pro5 MHC I H2Kb/SIINFEKL pentamer (ProImmune, UK) and excluding B cells. Frequency of iNKT cells was analyzed after staining with anti TCR-β (H57-597) and CD1d/αGalcer tetramer (kindly provided by F.Trottein, Institut Pasteur, Lille, France) and excluding B cells. IFN-γ intracellular staining was performed using the fixation/permeabilization kit from BD Biosciences. RORγt, Eomes and Tbet intracellular detection was performed using Foxp3 fixation/permeabilization kit from eBiosciences. Samples were run on a FACS Fortessa (BD Biosciences) and data were analyzed with Diva 6.1 (BD Biosciences) and FlowJo Version 10.7 (TreeStar) softwares.

### Immunofluorescence

Vagina mucosa and spleen were densified in a 30% sucrose solution, embedded in Shandon Cryomatrix ™ (ThermoElectron corp.) and frozen in a bath of isopentane cooled on dry ice. Tissue sections (7μm) were fixed in cold acetone and then saturated in PBS containing 0.1% Triton X-100, 0.05% tween 20, 2% mouse serum and 10% normal donkey serum. Mouse NKp46 and CD3ε stainings were performed using polyclonal goat anti-mouse NKp46 serum (R&D Systems) and monoclonal hamster anti-mouse CD3 antibody (2C11, BD Biosciences) after overnight incubation at 4°C. The day after, slides were incubated with Alexa594-conjugated donkey anti-goat antibody (Molecular Probes) to detect the NKp46 staining. After washes in PBS, slides were again saturated in PBS containing 0.1% Triton X-100, 0.05% tween 20, 2% mouse serum and 10% normal goat serum and incubated with Alexa488-conjugated goat anti-hamster antibody (Molecular Probes) to detect the CD3 staining. Slides were dried, mounted with Prolong Gold containing DAPI to stain nucleus and examined under Zeiss LSM 710 confocal microscope (Zeiss). Image processing was performed with Zeiss Zen and LSM softwares.

### Functional assays

Cells from spleen, GLN, liver, small intestine and vaginas were plated for 4h at 37°C in U-bottom 96 well plates and incubated with either PMA (200 ng/mL, Sigma-Aldrich) and ionomycin (0.5 μg/mL, Sigma-Aldrich) or mouse IL-12 (20 ng/mL, R&D Systems) and mouse IL-18 (5 ng/mL, MBL) or in the presence of YAC-1 tumor target cells at a 1:1 ratio or mouse IL-23 (20ng/ml) and mouse IL1β (20ng/ml). In addition, cells were incubated for 4h in high-affinity Immulon flat-bottom 96 well plates coated with 25 μg/mL of anti-mouse NK1.1 or anti-mouse IgG1 mAb. NK cell degranulation was assessed after CD107a staining and IFN-γ production evaluated in presence of monensin and brefeldin A (BD Biosciences).

### Statistics

Statistical analyses were carried out with Graphpad Prism software version 6.0 (GraphPad Prism, USA). Pairwise multiple comparisons of experimental groups were performed using the non parametric Mann-Whitney *U* test.

## Results

### Characterization of NKp46^+^ ILC in the mouse vagina

**Fig 1:**
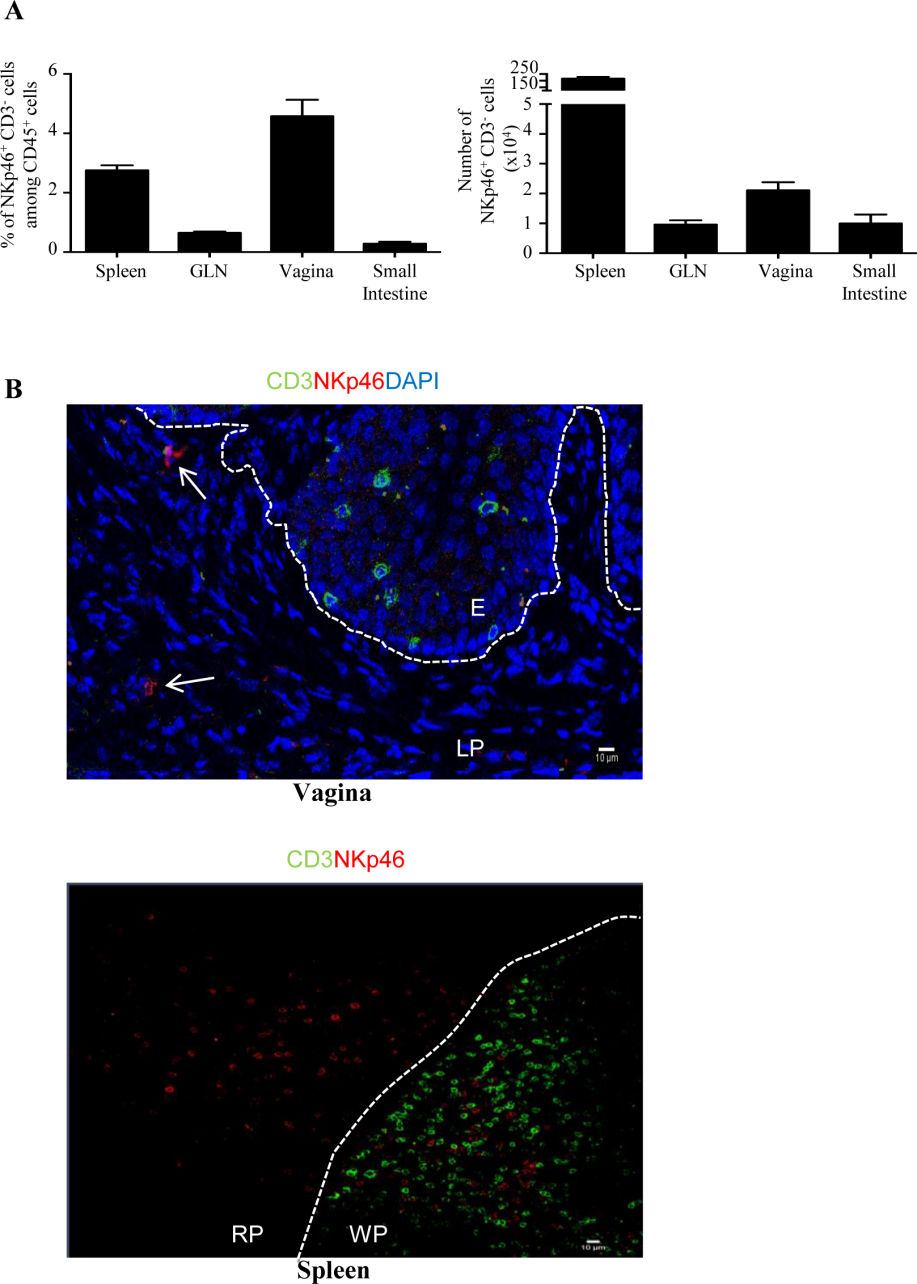
Identification of vaginal NKp46^+^ CD3^-^ cells. (A) Frequencies (left panel) and numbers (right panel) of NKp46^+^ CD3^-^ cells among CD45^+^ leukocytes from spleen, genital lymph nodes (GLN) and vagina of naive C57BL/6 mice. Histogram plots represent results from five independent experiments and are expressed as mean values + SEM, *n* = 10 mice. (B) Immunofluorescence staining of frozen sections from mouse vagina and spleen stained with anti-CD3 (green) and anti-NKp46 (red) antibodies. Nuclei were visualized with DAPI (blue). White arrows indicate NKp46^+^ CD3^-^ cells; E: epithelium; LP: lamina propria. WP: white pulp; RP: red pulp. White dotted lines delineate the epithelium from the lamina propria in the vagina and the white pulp from the red pulp in the spleen. Original magnification: x40 (vagina) and x20 (spleen). Data are representative of three independent experiments.

**Fig 2:**
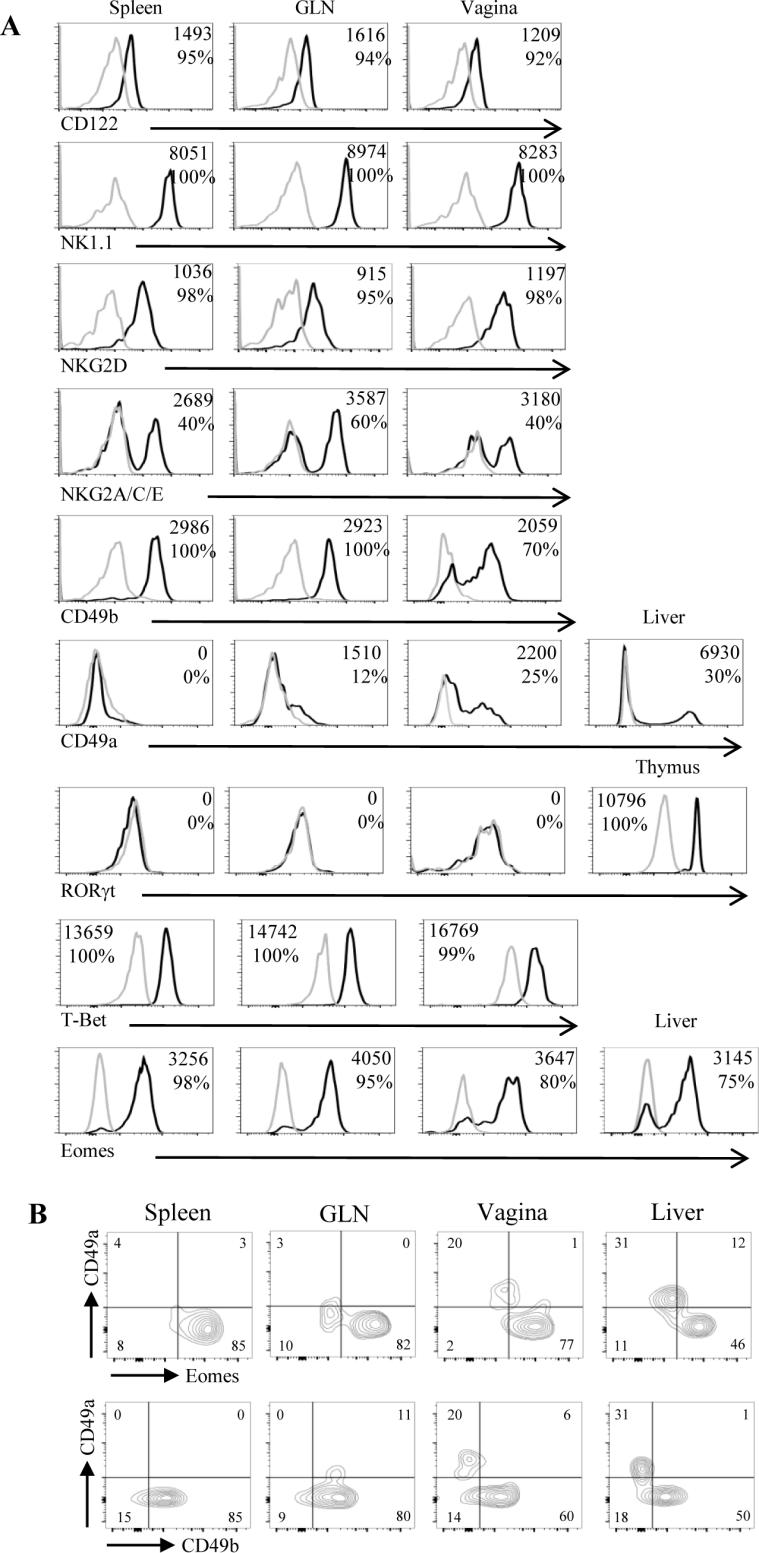
Vaginal NKp46^+^ CD3^-^ cell population contains conventional Eomes^+^ NK cells and Eomes^-^ ILC1-like cells. (A) Histogram plots show stainings with antibodies against specific markers (black line histograms) and with isotype control antibodies (grey line histograms) gated on NKp46^+^ CD3^-^ cells from spleen, GLN, and vagina of naive C57BL/6 mice. Specific stainings were also performed on NKp46^+^ CD3^-^ cells from liver and thymus. Numbers in histogram plots represent the MFI (mean fluorescence intensity) and the percentages of positive cells for the marker. Data are representative of at least four independent experiments. (B) Bidimensional dot plots show expression of CD49a and Eomes (upper panel) and CD49a and CD49b on gated NKp46^+^ CD3^-^ cells from spleen, GLN, vaginas and liver of naive C57BL/6 mice. Numbers in quadrants represent the percentage of cells for each subset.

**Fig 3:**
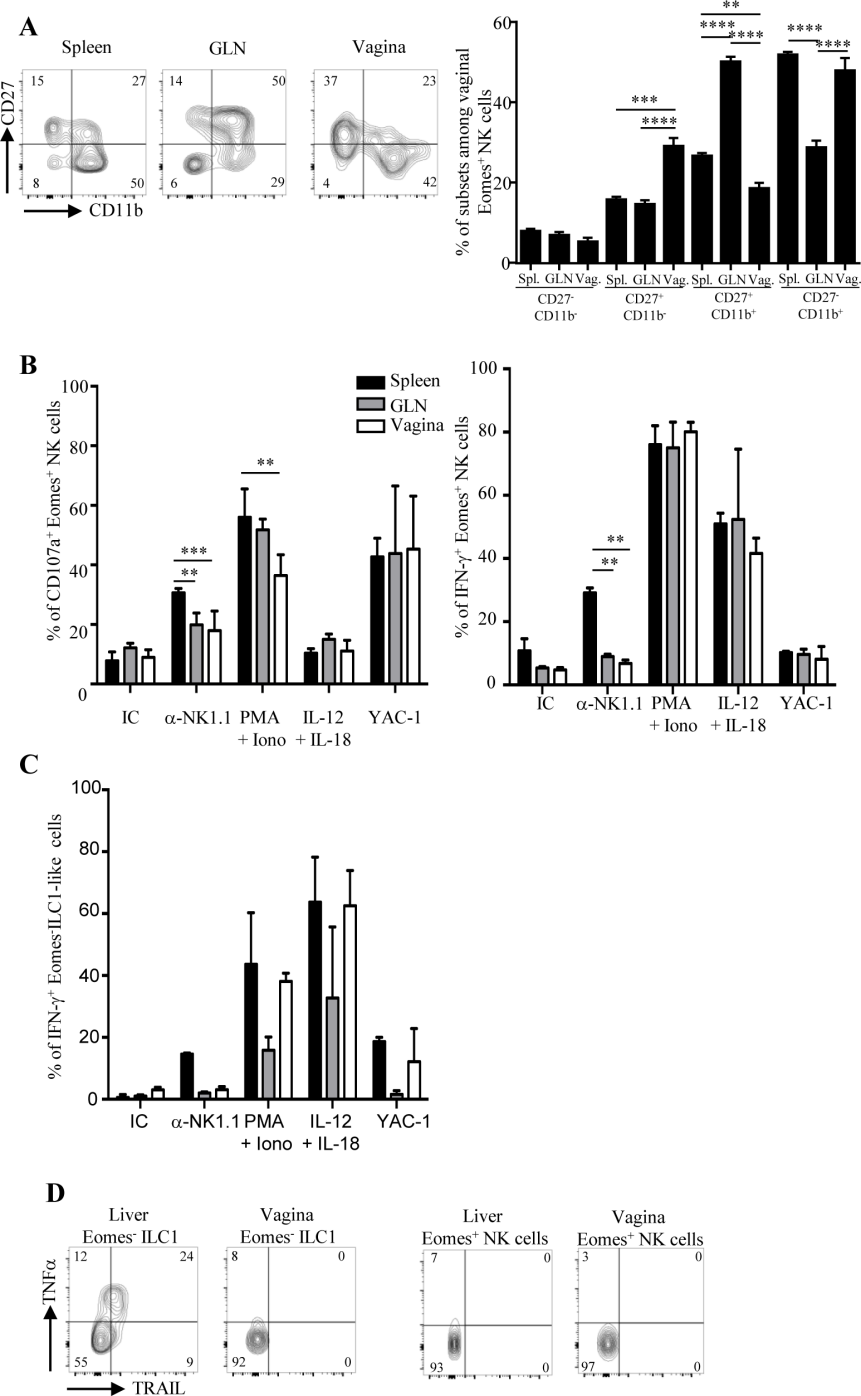
Functional properties of vaginal Eomes^+^ NK cells and Eomes^-^ ILC1-like cells. (A) Bidimensional dot plots (left panel) show expression of CD27 and CD11b on gated Eomes^+^ NKp46^+^ CD3^-^ cells from spleen, GLN and vaginas of naive C57BL/6 mice. Numbers in quadrants represent the percentage of cells for each subset. Histograms bars (right panel) represent results from five independent experiments expressed as mean values + SEM, *n* = 10 mice. **p<0.*01*, ***p<0.*001*, ****p<0.*0001* Mann-Whitney *U* test. Spl.: spleen; GLN: genital lymph nodes; Vag.: vagina. Frequencies of CD107a^+^ and IFN-γ^+^ producing Eomes^+^ NK cells (B) and Eomes^-^ ILC1-like cells (C) in cell suspensions from spleen, GLN and vagina of naive C57BL/6 mice. Cells (0.5 million/well) were stimulated *in vitro* for 4 hours with plate-bound isotype control (IC), anti NK1.1 mAb, PMA/ionomycin, IL-12 + IL-18 cytokines or YAC-1 tumor cells. CD107a and IFN-γ expression on cells were assessed by flow cytometry. Histogram bars represent results from three independent experiments expressed as mean percentages + SEM, *n* = 5 mice. **p<0.*01*, ***p<0.*001*, Mann-Whitney *U* test. (D) Bidimensional dot plots (left panel) show expression of TNFα and TRAIL on gated Eomes^-^ ILC1 and Eomes^+^ NK cells from liver and vaginas of naive C57BL/6 mice after stimulation *in vitro* for 4 hours with PMA/ionomycin. Numbers in quadrants represent the percentage of cells for each subset. Results are representative of two independent experiments with n = 4 mice.

While conventional NK cells and others ILC subsets were recently identified in various epithelial tissues, very little is known about the presence of these innate lymphoid cells in the vaginal tissue. We first identified a consistent proportion of NKp46^+^ CD3^-^ cells among CD45^+^ leukocytes in the vagina of naive C57BL/6 mice. These cells were more frequent among leukocytes of vagina (4.1 ± 0.4%) than among those of spleen (2.8 ± 0.2%), those of genital draining lymph nodes (GLN) (0.6 ± 0.1%) and those of small intestine (0.28 ± 0.05%) (Fig 1A left panel). The number of NKp46^+^ CD3^-^ cells was 2 fold higher in vagina compared to GLN (17 549 ± 2 735 and 8667 ± 1276, respectively) and small intestine (9993 ± 3000) but much lower than that of spleen (2.2.10^6^ ± 0.1. 10^6^) (Fig 1A right panel). To analyze the distribution of NKp46^+^ cells within the vaginal tissue, we also performed immunofluorescence staining on cryoconserved vaginal sections. NKp46^+^ CD3^-^ cells were detected in the lamina propria of the vaginal tissue (Fig 1B). Next, we measured the expression of various cell surface markers and transcription factors in order to determine whether vaginal NKp46^+^ CD3^-^ cells included conventional NK cells and/or subsets of ILC expressing NKp46. Vaginal NKp46^+^ CD3^-^ cells expressed CD122 (IL2Rβ), NK1.1 (NKR-P1C) and NKG2D homogeneously as well as CD94/NKG2A/C/E, and some Ly49 receptors with levels comparable to those seen on spleen and GLN NK cells (Fig 2A and S1 Fig). Moreover, they did not express CD117 and a fraction of them expressed CD127 (IL7Rα) (S1 Fig). Compared to splenic NKp46^+^ CD3^-^ cells, vaginal NKp46^+^ CD3^-^ cells displayed lower expression of CD49b (alpha 2 integrin) and a notable proportion of them expressed CD49a (alpha 1 integrin) indicating that the vaginal NKp46^+^ CD3^-^ cells population is heterogeneous and includes conventional CD49b^+^ NK cells and CD49a^+^ ILC1 [18]. We next analyzed the expression of several transcription factors *i*.*e* T-bet, Eomes and RORγt, known to be differentially expressed by NK cells, ILC1 and ILC3 subsets. Vaginal NKp46^+^ CD3^-^ cells were Tbet^+^ RORγt^-^, similarly as in the spleen and GLN. Furthermore, whereas most NKp46^+^ CD3^-^ cells expressed Eomes in the spleen and GLN, up to 20% of NKp46^+^ CD3^-^ cells were Eomes^-^ in the vagina (Fig 2A and 2B). This Eomes^-^ population specifically expressed CD49a but not CD49b, thus resembling the ILC1 subset previously identified in liver and intestine (Fig 2B). Nevertheless, unlike liver ILC1, vaginal NKp46^+^ CD3^-^ cells did not express TNF-related apoptosis-inducing ligand (TRAIL) (S1 Fig). Finally, based on the relative surface expression of CD27 and CD11b, mouse NK cells can be subdivided into four subsets with distinct functional properties: CD11b^-^CD27^-^ (precursor), CD11b^-^CD27^+^ (immature), CD11b^+^CD27^+^ (mature), CD11b^+^CD27^-^ (senescent) [23]. Vaginal Eomes^+^ NK cells had a significantly higher proportion of immature CD11b^-^CD27^+^ cells and a lower proportion of mature CD11b^+^CD27^+^ cells compared to spleen and GLN Eomes^+^ NK cells (Fig 3A).

We next compared the functional properties of Eomes^+^ NK cells and Eomes^-^ ILC1-like cells from vagina, spleen and GLN. We assessed their capacity to produce cytokines and to degranulate (measured by cell surface exposure of CD107a) following a 4h *ex vivo* stimulation assay by flow cytometry. Upon stimulation with PMA/Ionomycin, vaginal Eomes^+^ NK cells degranulated significantly less but produced similar amounts of IFN-γ compared to splenic and GLN Eomes^+^ NK cells (Fig 3B). In addition, upon crosslinking of the activating receptor NKR-P1C with the anti-NK1.1 mAb, vaginal Eomes^+^ NK cells degranulated significantly less and produced less IFN-γ than splenic Eomes^+^ NK cells. Finally, vaginal Eomes^+^ NK cells responded to stimulation with YAC-1 target cells, or with the IL-12 and IL-18 cytokine cocktail similarly as splenic and GLN Eomes^+^ NK cells (Fig 3B). In contrast, vaginal Eomes^-^ NKp46^+^ CD3^-^ ILC1-like cells did not degranulate. They also secreted IFN-γ but no TNF-α in response to different stimuli unlike liver ILC1 (Fig 3C and 3D and data not shown). More, vaginal NKp46^+^ cells did not produce IL-22 after *in vitro* stimulation contrary to intestinal NKp46^+^ RORγt^+^ ILC3 (S2 Fig).

Altogether, these results indicate that vaginal NKp46^+^ ILC population comprised a majority of conventional Eomes^+^ NK cells as well as an Eomes^-^ ILC1-like subset and both are functional after *ex vivo* stimulation.

### NKp46^+^ ILC limit vaginal CD8 T cell numbers generated by local immunization

**Fig 4:**
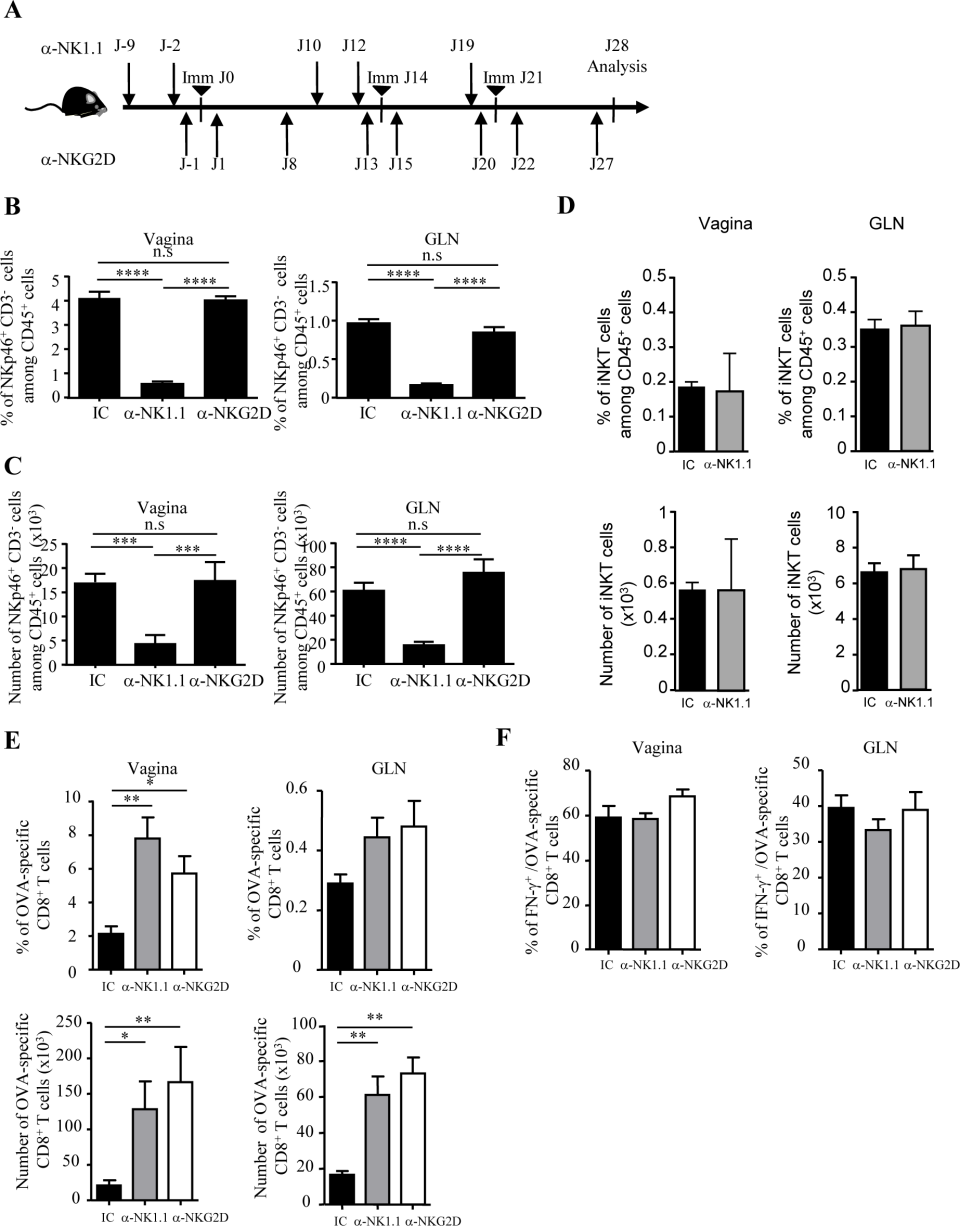
Vaginal NKp46^+^ ILC limit the number of effector CD8 T cells generated by vaginal immunization. (A) C57BL/6 mice received three intravaginal immunizations at days 0, 14, and 21 with the vaccine (CTBOVA+CT). Groups of mice received injections of either anti-NK1.1 ascite fluid (PK136), anti-NKG2D (CX5) mAb, or isotype-matched control mAb at the indicated days. At day 28, vaginas and GLN were collected and cell suspensions were analyzed by flow cytometry. (B, C and D) Frequencies and numbers of vaginal and GLN NKp46^+^ CD3^-^ cells and vaginal iNKT cells after NK1.1 depletion or NKG2D/NKG2D ligands blockade from vaccine-immunized C57BL/6 mice. Histogram bars represent results from three independent experiments expressed as mean values + SEM, n = 10 mice. (E) Frequencies and numbers of OVA-specific CD8 T cells in vagina and GLN upon vaccination. Cells were analyzed by flow cytometry at day 28 using OVA-specific MHC I pentamer, anti-CD3 and anti-CD8 mAbs. Histogram bars represent results from three independent experiments expressed as mean values + SEM, n = 10 mice. (F) Frequencies of IFN-γ^**+**^ OVA-specific CD8 T cells from vaginas and GLN after stimulation *in vitro* for 4 hours with PMA/ionomycin. IFN-γ expression on OVA-specific CD8 T cells was evaluated by flow cytometry after intracellular staining. Histogram bars represent results from three independent experiments expressed as mean values + SEM, n = 6 mice. *p<0.05, **p<0.01, ***p<0.01, ****p<0.001, n.s: no significatif, Mann-Whitney *U* test.

Next, we sought to evaluate the contribution of NKp46^+^ ILC on vaginal CD8 T cell responses induced by local vaccination. We used a well-characterized subunit vaccine composed of ovalbumin covalently linked to CTB and co-administered with CT (CTBOVA+CT called hereafter the vaccine) that generates vaginal OVA-specific CD8 T cells [6]. Female C57BL/6 mice were immunized at days 0, 14 and 21 by vaginal route with the vaccine (Fig 4A). The frequency of OVA-specific CD8 T cells was monitored 7 days after the last immunization in the vaginal mucosa and in the GLN. The impact of NKp46^+^ CD3^-^ cells on the vaginal CD8 T cell responses was determined in mice treated with a depleting antibody targeting the NK1.1 marker, homogeneously expressed by vaginal NKp46^+^ CD3^-^ cells (Fig 2A) This treatment efficiently depleted NKp46^+^ CD3^-^ cells in the vagina and GLN of vaccinated mice (Fig 4B and 4C; S3 Fig) without affecting vaginal CD1d/αGalCer^+^ iNKT cells known to express NK1.1 at lower level (Fig 4D). Remarkably, the depletion of NKp46^+^ CD3^-^ cells before each vaccination increased the frequency of vaginal OVA-specific CD8 T cells in the vagina by 3.7 fold compared to isotype control (anti NK1.1: 7.8 ± 1.2% vs. IC: 2.1 ± 0.5%) as well as their numbers by 6.2 fold (anti NK1.1: 128 259 ± 39 242 vs. IC: 20 605 ± 7 410) (Fig 4E). In GLN, we also observed a 3.7 fold increase in the number of OVA-specific CD8 T cells (anti NK1.1: 61 260 ± 10 200 vs. IC: 16 500 ± 2 190). The frequency and numbers of non specific CD8 T cells as well as the frequency of IFN-γ producing OVA-specific CD8 T cells were not affected by the depletion of NK cells in the vagina and in the GLN (Fig 4F and data not shown).

These results indicate that NKp46^+^ ILC negatively regulate the pool of vaginal antigen-specific CD8 T cells induced upon local immunization without affecting their effector functions.

### The NKG2D/NKG2D ligand interaction is involved in the regulation of vaccine-induced CD8 T cells after vaginal immunization

**Fig 5:**
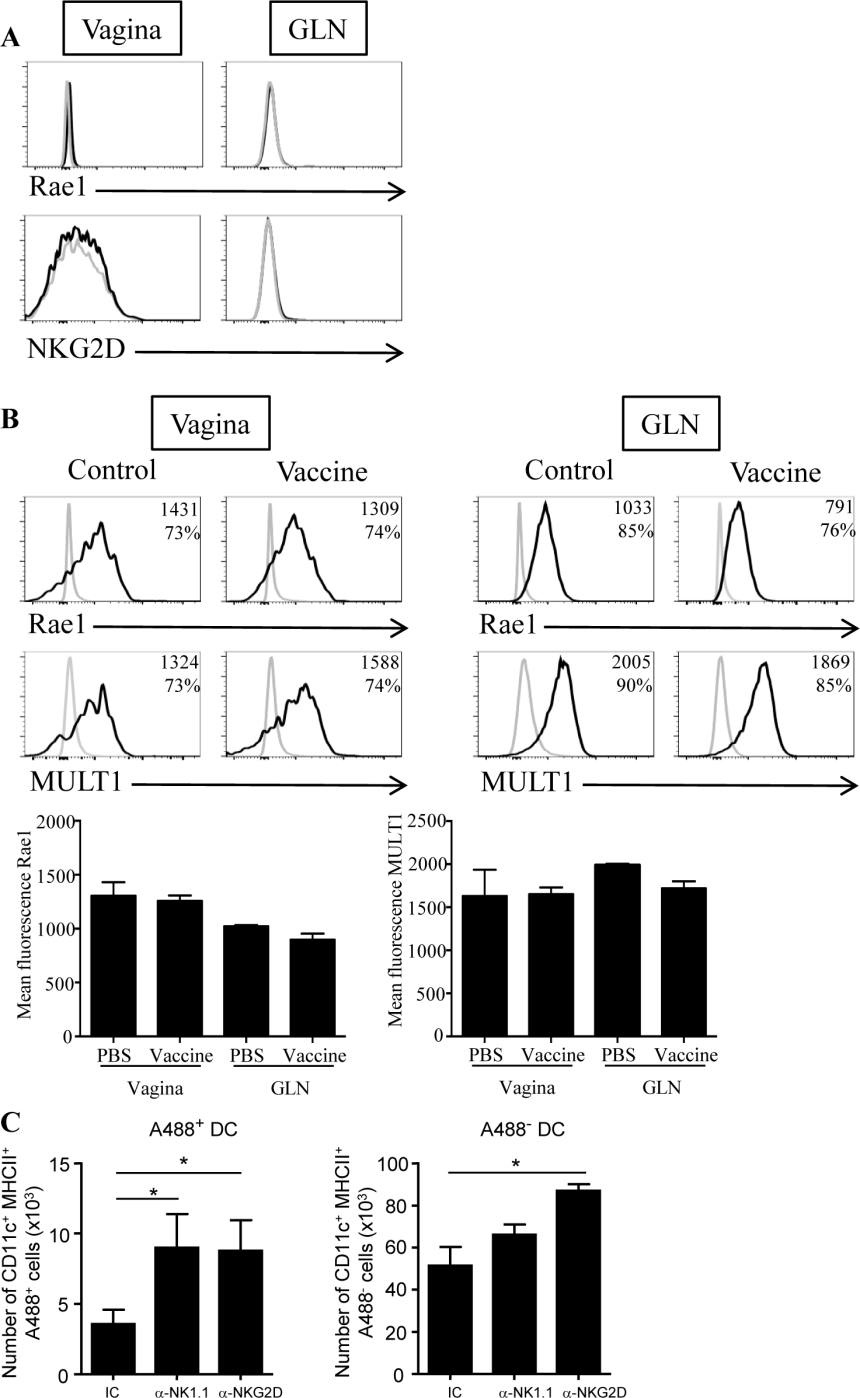
NKG2D/NKG2D ligands interaction control vaginal dendritic cell numbers after local immunization. (A) Naive C57BL/6 mice received three intravaginal immunizations at days 0, 14, and 21 with the vaccine (CTBOVA+CT). At day 28, cells from vaginas and GLN were analyzed by flow cytometry to assess the expression of NKG2D and the NKG2D ligand Rae1 on OVA- specific CD8 T cells. Histogram plots show stainings with antibodies against specific markers (black line) and with isotype control antibodies (grey line). (B) Dendritic cells from vagina (left panel) and GLN (right panel) of vaccine or PBS-treated mice were prepared 7 days after immunization with the vaccine and were analyzed for expression of the NKG2D ligands Rae1 and MULT1 by flow cytometry. Numbers in histograms plots represent the mean fluorescence intensity (MFI) and the percentages of positive cells for the marker. Data are representative of 2 independent experiments. Histogram bars represent results from 2 independent experiments expressed as mean values + SEM, n = 5 mice. (C) After immunization with CTBA488+CT, the number of antigen-bearing dendritic cells (A488^+^ CD11c^+^ MHCII^+^) and other DC (A488^-^ CD11c^+^ MHCII^**+**^) were analyzed at day 7 in the vagina of mice treated with anti-NK1.1 ascite fluid, isotype control and anti-NKG2D antibodies. Results from three independent experiments are expressed as mean numbers +SEM, n = 6. *p<0.001, Mann-Whitney *U* test.

NK cells were already described to limit effector and/or memory CD8 T cell responses in mouse models of LCMV infection or systemic immunization by a mechanism involving the activating receptor NKG2D [24, 25]. As vaginal NKp46^+^ ILC homogeneously expressed NKG2D (Fig 2A), we then addressed its contribution in the regulation of vaginal CD8 T cell responses. C57BL/6 mice were treated with the blocking anti-NKG2D mAb (clone CX5) throughout the immunization protocol (Fig 4A). This treatment did not affect the numbers of NKp46^+^ CD3^-^ cells in vagina and in GLN (Fig 4B and 4C). Similarly to anti-NK1.1 treatment, NKG2D blockade significantly increased the number of OVA-specific CD8 T cells by 8 fold in the vagina and by 4.4 fold in GLN but did not affect IFN-γ production by OVA-specific CD8 T cells (Fig 4E and 4F). Upon activation, it was previously shown that CD8 T cells can express NKG2D and NKG2D ligands such as Rae1 [26, 27]. Nevertheless, in our vaccination model, antigen-specific CD8 T cells did not express NKG2D or its ligand Rae1, excluding their NKG2D-dependent elimination directly by vaginal NKp46^+^ ILC or by NKG2D^+^ CD8 T cells (Fig 5A). Consequently, this suggested that vaginal NKp46^+^ ILC control CD8 T cell effectors indirectly after local vaccination. We next measured the expression of NKG2D ligands on vaginal dendritic cells as they are responsible for the generation of OVA-specific CD8 cells after local vaccination with the vaccine [6, 8]. Vaginal and GLN dendritic cells from immunized and control mice expressed substantial levels of the NKG2D ligands Rae1 and MULT1 whereas T cells did not (Fig 5B and S4 Fig), suggesting that they can be target of NKG2D^+^ NK cells.

Taking into account that vaginal DC but not CD8 T cells expressed NKG2D ligands, we next assessed whether the depletion of NKp46^+^ cells or NKG2D blockade affected the number of vaginal DC. NKp46^+^ cells peaked at day 6 after vaginal administration of the vaccine (S5 Fig) suggesting that the maximal impact on DC frequency should be observed one day later. We monitored the number of vaginal CD11c^+^ MHCII^+^ DC 7 days after one immunization using CTB coupled to the Alexa488 fluorescent tracer in WT and NKp46^+^ ILC depleted mice. A488^+^ DC allowed the tracking of DC that had captured the antigen at the site of immunization. Interestingly, after NKp46^+^ ILC depletion, the number of antigen-bearing DC in vagina significantly increased by 2.5 fold (A488^+^ DC: 8 983 ± 2 398 vs. 3 583 ± 1 005) while the number of DC that did not capture the antigen (*i*.*e*. A488^-^ DC) was not significantly affected (66 050 ± 4 824 vs. 51 346 ± 8 873) (Fig 5C). NKp46^+^ ILC depletion had no effect on GLN DC numbers (data not shown). Finally, the blockade of NKG2D/NKG2D ligands interaction induced an increase in the number of antigen-bearing DC in vagina compared to control group (A488^+^ DC: 8 764 ± 2 173 vs. 3 586 ± 1 005). This blockade also induced a significant increase in the number of vaginal A488^-^ DC (DC: 87 011 ± 3 167 vs. 51 346 ± 8 873). Nevertheless, we cannot exclude that the expansion of effector cells in the vagina also contribute to the recruitment of vaginal DC.

Altogether, these results demonstrate that either NKp46^+^ ILC depletion or blockade of the interaction between NKG2DL^+^ DC and NKG2D^+^ NKp46^+^ ILC similarly contribute to the regulation of vaginal CD8 T cell responses. This suggests that cytotoxic NKp46^+^ ILC indirectly control vaccine-induced CD8 T cell responses through their interaction with antigen-bearing dendritic cells.

## Discussion

In this study, we characterize NKp46^+^ CD3^-^ ILC in the mouse vagina and show that this subset contains conventional Eomes^+^ NK cells and Eomes^-^ ILC1-like cells. Our data demonstrate that NKp46^+^ CD3^-^ ILC dampen vaginal CD8 T cell response to a local vaccine by an indirect NKG2D-dependent mechanism affecting numbers of vaginal dendritic cells.

The ILC1 family includes the well-described conventional NK cells and ILC1 subsets recently identified in liver, intestine, skin, thymus and uterus [22, 28–31].They are closely related as they express the transcription factor T-bet, NK cell receptors such as NKp46, NKG2D and NK1.1 and produce IFN- γ. Conventional NK cells and subsets of ILC1 were first discriminated on the basis of different functional properties, NK cells being “cytotoxic” cells while ILC1 were rather “helper-like” cytokine producers. Subsets of ILC1 are also described to be endowed with cytotoxic properties via TRAIL or indirectly via secretion of TNF-α or IFN-γ [18, 32]. Consequently, Serafini et al., proposed to distinguish conventional NK and ILC subsets on the basis of Eomes expression with conventional NK being Eomes^+^ and other ILC being Eomes^-^ [18]. In the mouse vagina, we were able to identify conventional NK cells and ILC1-like cells. To our knowledge, this is the first characterization of conventional NK and ILC1 in such epithelial tissue. Nevertheless, we cannot rule out the possibility that vaginal NKp46^+^ Eomes^-^ cells also include a subset of ex-ROR-γt ILC3 that differentiated into ILC1 by downregulation of RORγt and upregulation of T-bet [29, 33–35]. Further fate mapping experiments will be necessary to fully characterize mouse vaginal ILC. Vaginal ILC1 do not share all the features of liver ILC1, as they do not express TRAIL and do not produce TNF-α after *in vitro* stimulation. This suggests that the vaginal epithelial environment favors the emergence of a peculiar ILC1 subset. Comparative gene expression analyzes may help to fully characterize this new subset of ILC1 as well as to decipher the molecular events involved in the muco-epithelial imprinting of this subset of ILC1.

Vaginal Eomes^+^ NK cells have a higher frequency of the immature CD11b^-^CD27^+^ subset as well as a lower expression of markers associated with NK cell maturation (CD49b and CD43) than splenic Eomes^+^ NK cells [36, 37]. The higher frequency of immature Eomes^+^ NK cells in the vagina can explain their lower effector functions after *in vitro* stimulation. These results are consistent with other studies that have demonstrated that NK cells from skin and gut exhibit lower effector functions than NK cells from secondary lymphoid organs [16, 17, 38]. Altogether, this suggests that the naturally tolerogenic environment of *epithelia* controls inappropriate NK cell functions and reactivity in steady conditions.

Many studies have shown that NK cells impair T cell responses after systemic immunization or viral infection [39, 40]. Several mechanisms whereby NK cells regulate T cell responses were reported including engagement of the activating receptor NKG2D, TRAIL-dependent apoptosis, or cytokine production by NK cells [25, 37, 41–47]. Our study extends these observations to the vaginal compartment and indicates that vaginal NKp46^+^ ILC are negative regulators of vaccine-induced CD8 T cell responses. The respective contribution of conventional NK cells and ILC1 subsets in this phenotype remains to be established. Indeed, some features of conventional NK cells are shared by ILC1. In the liver and the salivary glands, NKp46^+^ ILC1 subset secretes cytokines such as TNF-α, IFN-γ and IL-2 upon *in vitro* or *in vivo* stimulation and also expresses NKG2D and TRAIL [28, 48, 49]. In the intestine, subsets of ILC1 are also cytokine producers (IFN-γ and TNF-α) after *in vitro* stimulation with IL-12 + IL-15 or IL-12+ IL-18 or after mucosal infection [22, 29, 50].They can also also degranulate after *in vitro* stimulation in the presence of tumor cell line [22]. In the vagina, we showed that NKp46^+^ Eomes^-^ ILC1 also secrete IFN-γ after *in vitro* stimulation but did no degranulate in response to various stimuli, suggesting that vaginal ILC1 are mainly cytokine producers contrary to NK cells. Consequently, one may expect that NK cells rather than ILC1 are the negative regulators of vaccine-induced CD8 T cell responses. Nevertheless, how the vaccine modulates the functional properties of both subsets *in vivo* remains to be formely established.

In that sense, upon infection or during inflammation, NKp46^+^ ILC have complementary roles in host-protective responses. The role of conventional NK cells in killing microbe-infected cells have been extensively described [14]. During skin inflammation or viral infection, liver NKp46^+^ cells mediate specific recall responses [51–53]. More, Peng et al. demonstrated that liver-resident CD49a^+^ CD49b^-^ NK1.1^+^ cells, presumably ILC1, are the cells that contribute to skin inflammation [32]. More, IFN-γ producing ILC1 confer protection against intestinal *Salmonella enterica* and *Toxoplasma gondii* infection [29, 35].

NKG2D or TRAIL receptors have been involved in the lysis of activated CD4, CD8 T cells or DC [24, 49, 54]. Our data suggest that local NKp46^+^ ILC shape vaginal CD8 T cell responses through a NKG2D-dependent mechanism regulating DC numbers. The observation that vaginal ILC1-like cells cannot degranulate in response to various stimuli *in vitro* contrary to conventional NK cells strongly suggest that conventional NK cells rather than ILC1 dampen vaginal CD8 T cell responses. Nevertheless, we cannot exclude that vaginal NKp46^+^ Eomes^-^ ILC1contribute to this phenotype by the production of helper cytokines. In fact, liver Eomes^-^ ILC1 are described to produce huge amounts of IL-2 upon *in vitro* or *in vivo* stimulation, a cytokine known to activate conventional NK cells [28]. Finally, in line with our results, other studies have shown that microbe-infected DC expressing NKG2D ligands are targets of NK cell lysis [54, 55] More, it was also proposed that contaminating NK cells in co cultures between DC and CD4 T cells reduce the ability of vagina-derived DC to prime CD4 T cells [56]. Our data highlight that the fine tune dialog between vaginal ILC and DC, two immune cell types present in the genital tract, impacts the amplitude of mucosal CD8 T cell responses.

In conclusion, our results reveal an unappreciated role of vagina-associated NKp46^+^ ILC in the regulation of vaccine-induced CD8 responses after local immunization with a subunit vaccine. Determining how to manipulate this regulatory activity at the cellular and molecular levels will help to improve vaccines efficacy.

## Supporting Information

S1 Fig Phenotype of vaginal NKp46^+^ CD3^-^ ILC. Surface or intracellular expression of indicated markers analyzed by flow cytometry on gated NKp46^+^ CD3^-^ cells from spleen, GLN, vagina, thymus of naive C57BL/6 mice. Histogram plots show stainings with antibodies against specific markers (dark grey histograms) and with isotype control antibodies (white histograms). Numbers in histograms plots represent the MFI (mean fluorescence intensity) and the percentages of positive cells. Cells were preincubated with anti FcγRII/III mAb (2.4G2) for 10 min at 4°C to block Fc receptors. Unless indicated, all antibodies were purchased from BD Biosciences. Cells were analyzed by using the following mAb: Ly49D (4E5), Ly49A/D (12A8), Ly49G2 (4D11), KLRG1 (2F1), CD43 (S7), CD117 (2B8), CD127 (A7R34), TRAIL (N2B2, eBiosciences), CD69 (H1.2F3), Samples were run on a FACS Fortessa (BD biosciences) and data e analyzed with Diva6.1 (BD biosciences) and FlowJo Version10.7 softwares (TreeStar).
(TIF)

S2 Fig NKp46^+^ CD3^-^ ILC did not produce IL-22 in the vagina unlike in the small intestine. Bidimensional dot plots (left panel) show expression of IL-22 and RORγt on gated CD3^-^ NKp46^+^ from small intestine and vaginas of naive C57BL/6 mice after stimulation *in vitro* for 4 hours with IL-23 (20ng/ml) + IL-1β (20ng/ml). Numbers in quadrants represent the percentage of cells for each subset. Results are representative of two independent experiments with n = 4 mice.
(TIF)

S3 Fig Depletion of vaginal NKp46^+^ CD3^-^ ILC. C57BL/6 mice received three intravaginal immunizations at days 0, 14, and 21 with the CT-based vaccine (CTBOVA+CT). Groups of mice received injections of either anti-NK1.1 ascite fluid (PK136), or isotype-matched control antibody at day -9 and 2 days before each immunization. Dot plot FACS profiles show the frequency of NKp46^+^ CD3^-^ ILC in isotype-matched control and anti-NK1.1 treated mice in the vagina and in the GLN.
(TIF)

S4 Fig Expression of NKG2D ligands on vaginal and DLN T cells of immunized mice. T lymphocytes from vaginas (left panel) and GLN (right panel) of vaccine-treated mice were analyzed for expression of the NKG2D ligands Rae1 and MULT1 by flow cytometry. Numbers in histograms plots represent the mean fluorescence intensity (MFI) and the percentages of positive cells for the marker. Data are representative of 2 independent experiments.
(TIF)

S5 Fig Vaginal NKp46 ILC expand after local immunization with CTBOVA+CT. C57BL/6 mice were immunized intravaginally with the vaccine (CTBOVA+CT). Cell suspensions from vagina of vaccine or PBS-treated mice were analyzed by flow cytometry at different time points after vaccination to determine NKp46 cell numbers. Histogram bars represent results from three independent experiments expressed as mean values + SEM, *n* = 4–10 mice. ****p<0.*0001*, ***p<0.*001*, **p<0.*01*, *p<0.*05;* Mann-Whitney test.
(TIF)
