# Supplementary figures and images for: NKp46+ Innate Lymphoid Cells Dampen Vaginal CD8 T Cell Responses following Local Immunization with a Cholera Toxin-Based Vaccine
Source: PLoS One. 2015 Dec 2;10(12):e0143224. doi: 10.1371/journal.pone.0143224 (PMC4668070; doi:10.1371/journal.pone.0143224)

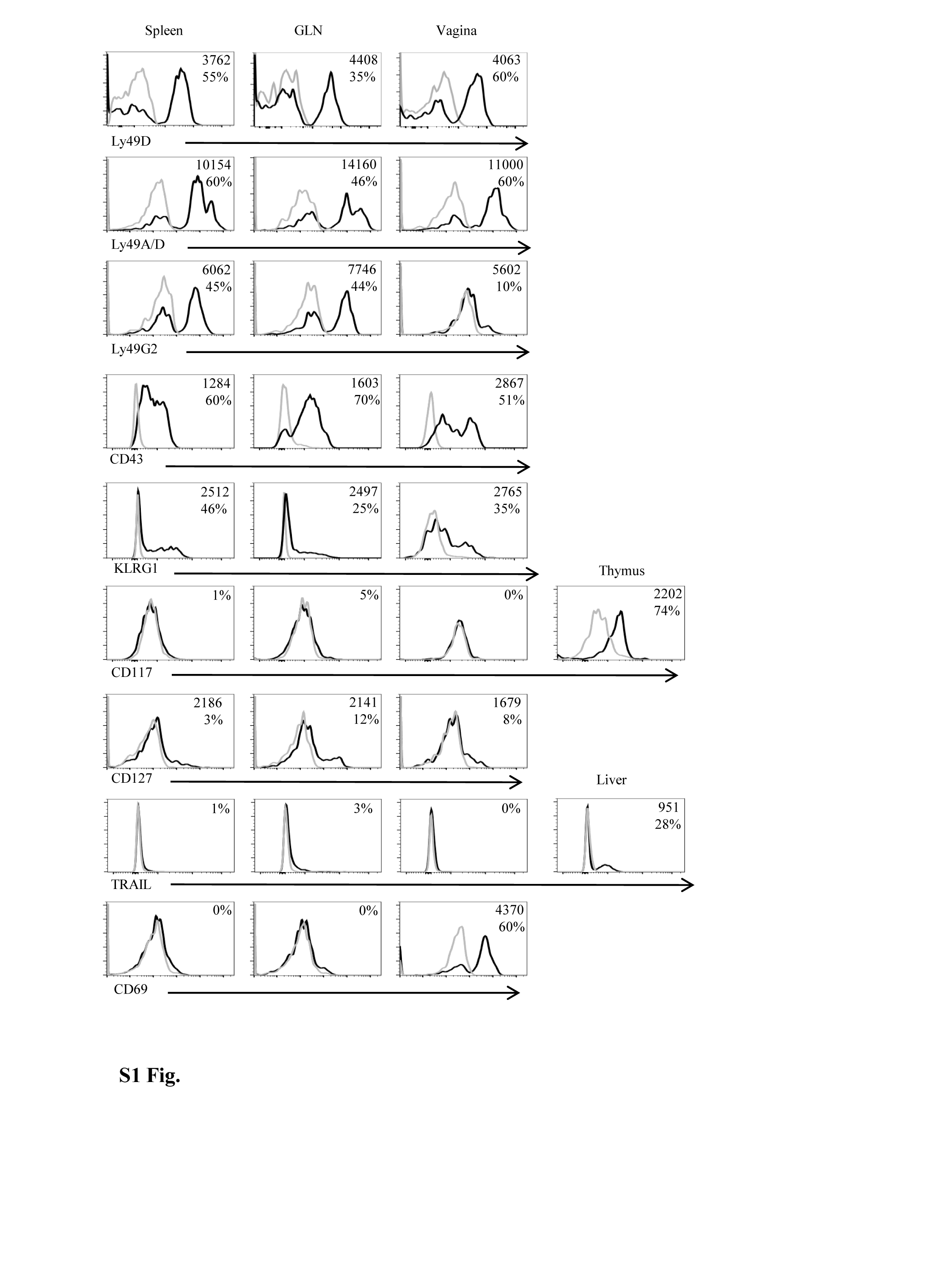

Supplement: S1 Fig — Surface or intracellular expression of indicated markers analyzed by flow cytometry on gated NKp46+ CD3- cells from spleen, GLN, vagina, thymus of naive C57BL/6 mice. Histogram plots show stainings with antibodies against specific markers (dark grey histograms) and with isotype control antibodies (white histograms). Numbers in histograms plots represent the MFI (mean fluorescence intensity) and the percentages of positive cells. Cells were preincubated with anti FcγRII/III mAb (2.4G2) for 10 min at 4°C to block Fc receptors. Unless indicated, all antibodies were purchased from BD Biosciences. Cells were analyzed by using the following mAb: Ly49D (4E5), Ly49A/D (12A8), Ly49G2 (4D11), KLRG1 (2F1), CD43 (S7), CD117 (2B8), CD127 (A7R34), TRAIL (N2B2, eBiosciences), CD69 (H1.2F3), Samples were run on a FACS Fortessa (BD biosciences) and data e analyzed with Diva6.1 (BD biosciences) and FlowJo Version10.7 softwares (TreeStar). (TIF) [file pone.0143224.s001.tif]

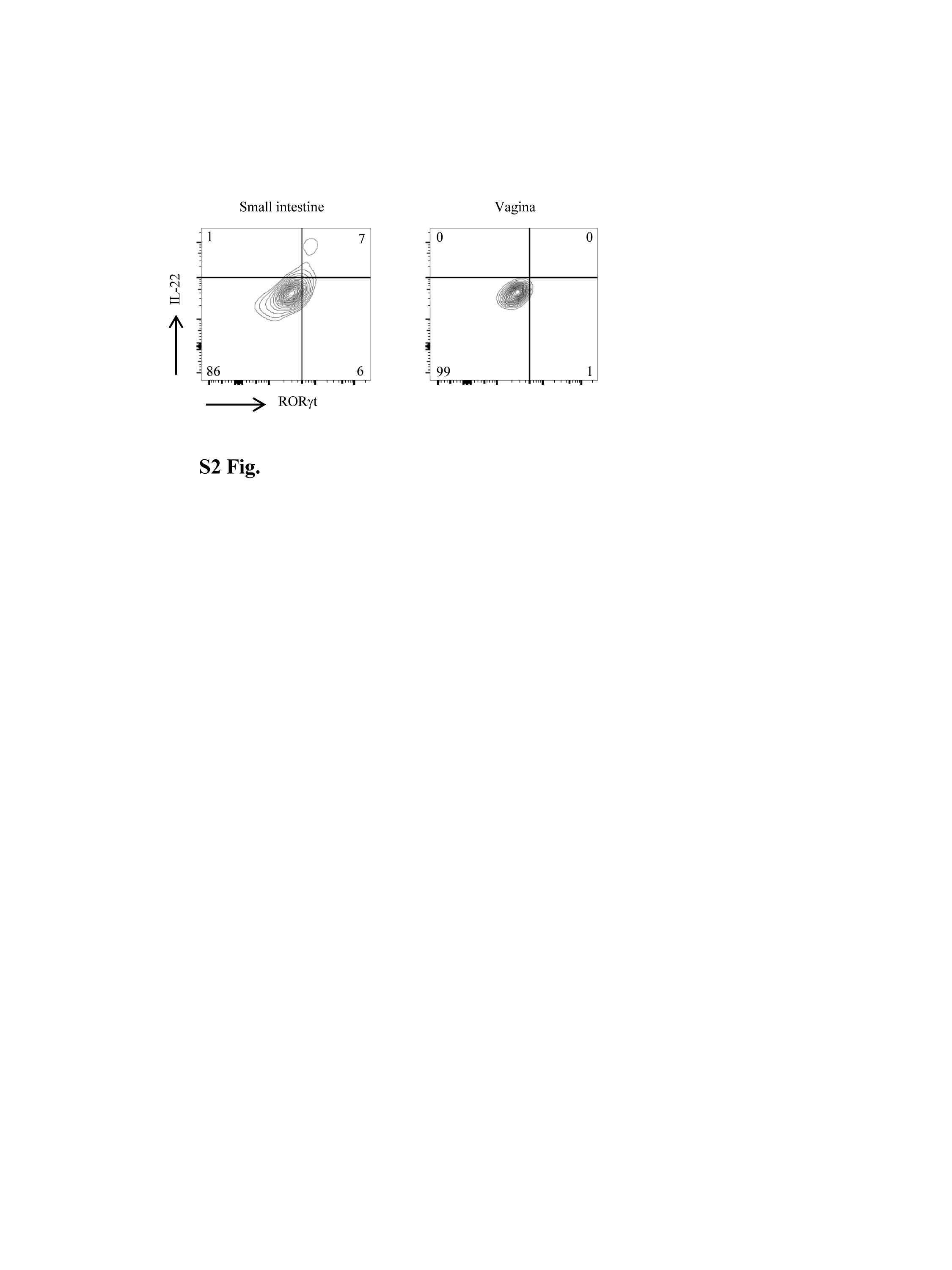

Supplement: S2 Fig — Bidimensional dot plots (left panel) show expression of IL-22 and RORγt on gated CD3- NKp46+ from small intestine and vaginas of naive C57BL/6 mice after stimulation in vitro for 4 hours with IL-23 (20ng/ml) + IL-1β (20ng/ml). Numbers in quadrants represent the percentage of cells for each subset. Results are representative of two independent experiments with n = 4 mice. (TIF) [file pone.0143224.s002.tif]

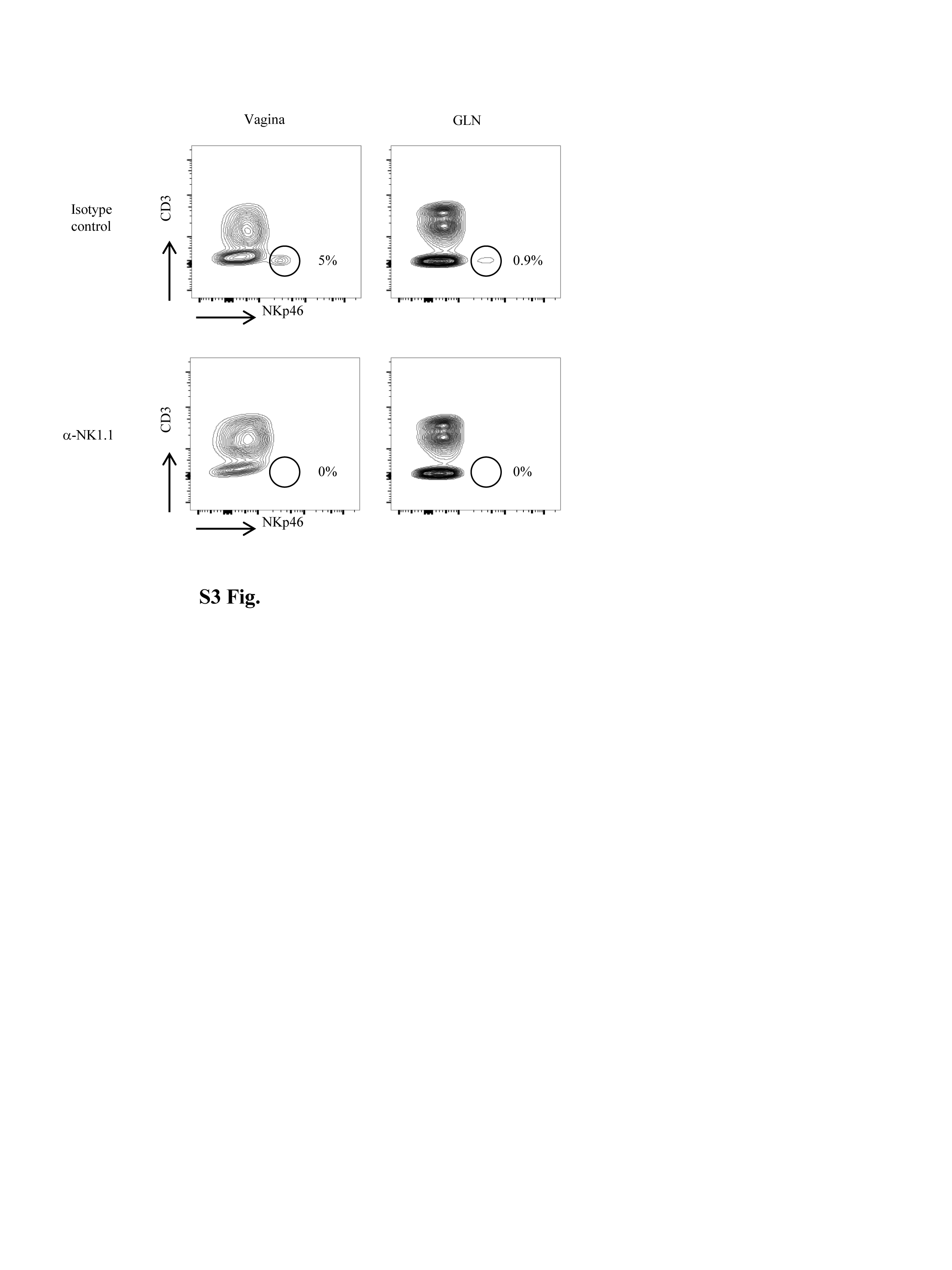

Supplement: S3 Fig — C57BL/6 mice received three intravaginal immunizations at days 0, 14, and 21 with the CT-based vaccine (CTBOVA+CT). Groups of mice received injections of either anti-NK1.1 ascite fluid (PK136), or isotype-matched control antibody at day -9 and 2 days before each immunization. Dot plot FACS profiles show the frequency of NKp46+ CD3- ILC in isotype-matched control and anti-NK1.1 treated mice in the vagina and in the GLN. (TIF) [file pone.0143224.s003.tif]

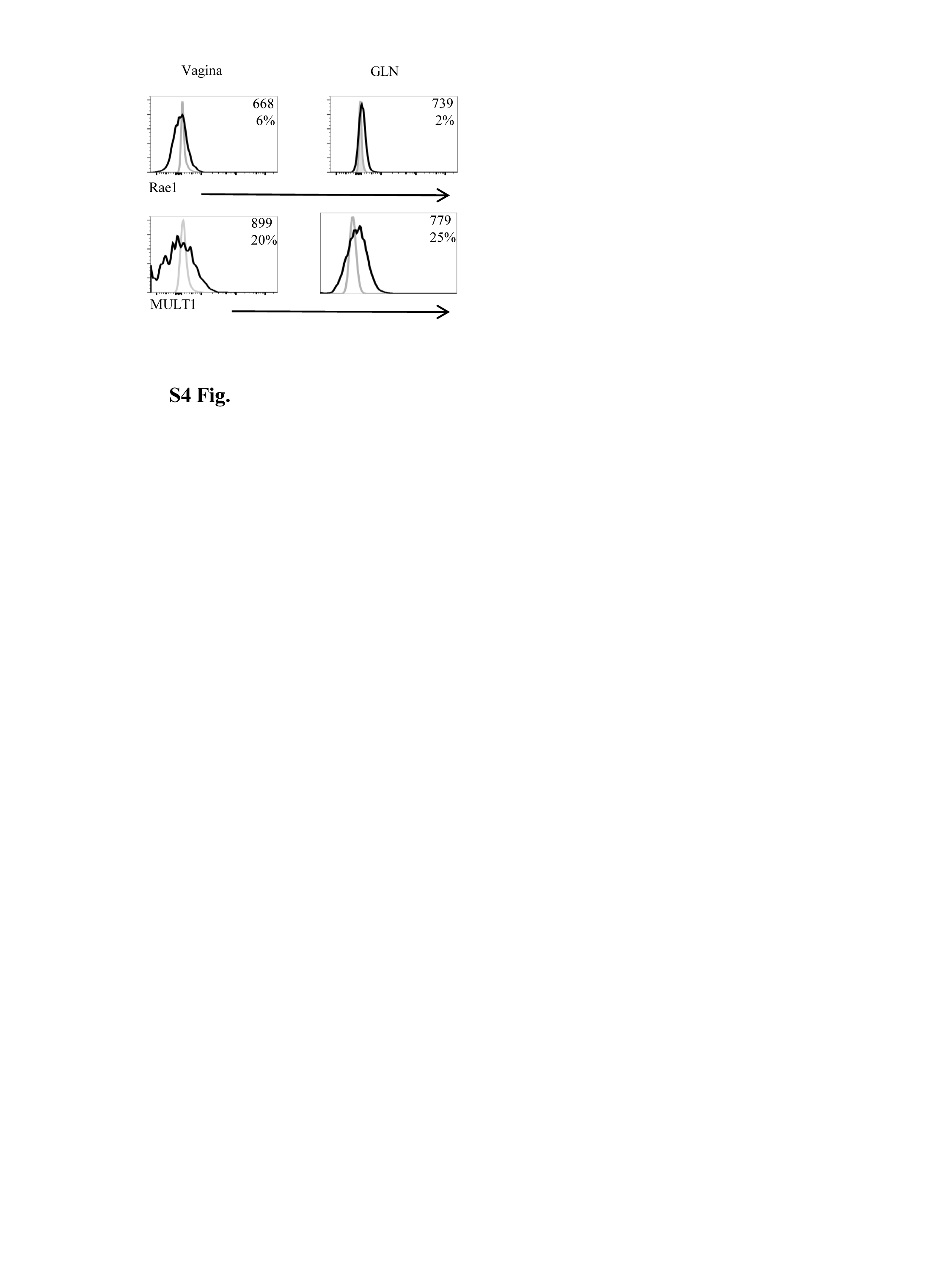

Supplement: S4 Fig — T lymphocytes from vaginas (left panel) and GLN (right panel) of vaccine-treated mice were analyzed for expression of the NKG2D ligands Rae1 and MULT1 by flow cytometry. Numbers in histograms plots represent the mean fluorescence intensity (MFI) and the percentages of positive cells for the marker. Data are representative of 2 independent experiments. (TIF) [file pone.0143224.s004.tif]

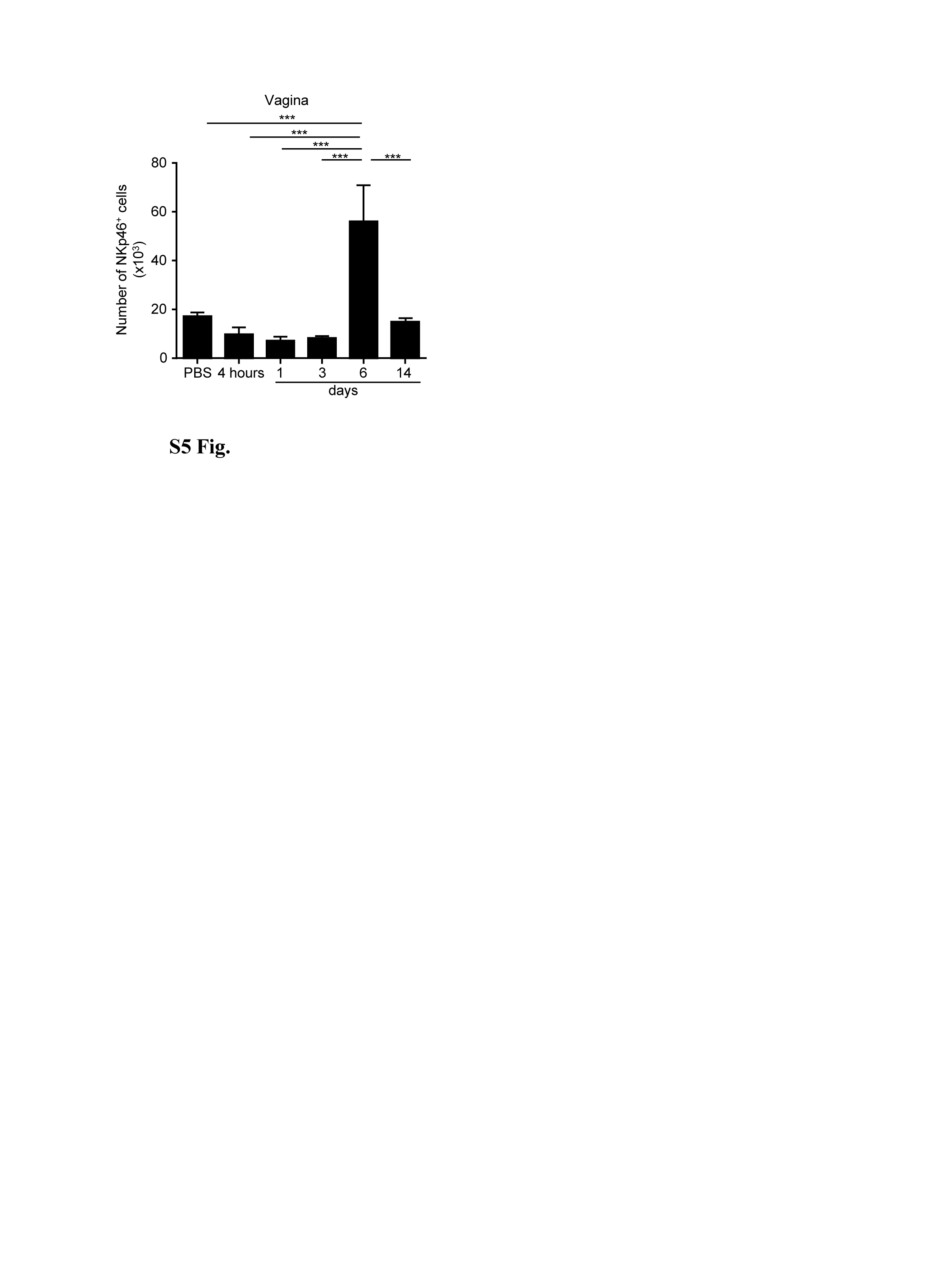

Supplement: S5 Fig — C57BL/6 mice were immunized intravaginally with the vaccine (CTBOVA+CT). Cell suspensions from vagina of vaccine or PBS-treated mice were analyzed by flow cytometry at different time points after vaccination to determine NKp46 cell numbers. Histogram bars represent results from three independent experiments expressed as mean values + SEM, n = 4–10 mice. ****p<0.0001, ***p<0.001, **p<0.01, *p<0.05; Mann-Whitney test. (TIF) [file pone.0143224.s005.tif]
